# The phytocannabinoid cannabidivarin alleviates cognitive and social behaviour deficits in the sub-chronic phencyclidine rat model of relevance for schizophrenia

**DOI:** 10.1177/02698811251381246

**Published:** 2025-10-29

**Authors:** Ben Grayson, Giovanni Podda, Jackie Cilia, Marie Woolley-Roberts, Joanna C. Neill, Jennifer Fletcher, Michael Harte

**Affiliations:** 1 Division of Pharmacy and Optometry, School of Health Sciences, Faculty of Biology, Medicine and Health, University of Manchester, UK; 2 Jazz Pharmaceuticals Research UK Ltd, Sittingbourne, UK

**Keywords:** CBDV, schizophrenia, sub-chronic PCP, phytocannabinoids, cognition, novel object recognition, social deficits, reversal learning

## Abstract

**Background:**

Schizophrenia is a serious psychiatric disorder that affects over 24 million people worldwide. Current therapies treat positive symptoms but show little efficacy for negative and cognitive symptoms, which can impact quality of life. Development of effective medication for this unmet medical need is imperative. Phytocannabinoids, such as cannabidivarin (CBDV), show promising results for the treatment of neurological disorders and have demonstrated efficacy in preclinical models, including seizure, autism, Rett, and Fragile X syndromes.

**Aim:**

To determine the effect of CBDV on cognitive and social deficits in a sub-chronic phencyclidine (scPCP) rat model of relevance to schizophrenia.

**Methods:**

Female Lister Hooded rats received scPCP (2 mg/kg, intraperitoneally (i.p.)) or saline for 7 days, followed by a minimum 7-day drug-free washout. Rats were then treated with saline, CBDV (2, 10, and 20 mg/kg, i.p.), or risperidone (0.1 mg/kg, i.p.) 60 minutes before novel object recognition (NOR), social interaction (SI), and reversal learning (RL) testing.

**Results:**

scPCP significantly impaired performance in all three behavioural tests. Deficits in the NOR and SI tests were significantly improved by CBDV (10 and 20 mg/kg), while 20 mg/kg was effective at reversing the deficits in RL. Risperidone also significantly reversed the scPCP-induced deficit in the NOR, RL, and SI paradigms.

**Conclusion:**

Here, we are the first to demonstrate the ability of CBDV to ameliorate cognitive and social deficits in the scPCP rat model of relevance for schizophrenia, suggesting further research is justified into the potential therapeutic benefits of CBDV in patients.

## Introduction

Cognitive deficits and negative symptoms remain an unmet clinical need in schizophrenia (Citrome, 2014). As a result, patients with schizophrenia have a significantly impaired quality of life (Walker et al., 2017). This comes at a considerable cost, not only to patients and carers but also to the economy. The most recent figures, based on a search of published research from 2006 to 2021 in the United States, United Kingdom, France, Germany, Italy, Spain, Canada, Japan, Brazil, and China, estimate that schizophrenia has the highest median societal cost per patient of all mental disorders (Kotzeva et al., 2023) and is one of the leading causes of disability worldwide (Chong et al., 2016). Several novel targets, such as encenicline, a partial agonist at nicotinic α7 receptors and bitopertin, a glycine transporter-1 inhibitor, have been tested for the alleviation of these symptoms. However, the failure in large clinical trials of these novel compounds illustrates the urgent need for radical new approaches (see Shahid et al., 2020 for an overview).

Recently, chemicals extracted from *Cannabis sativa* L., such as phytocannabinoids, have been proposed as novel treatments for psychiatric disorders, including schizophrenia (Noel, 2017). The complexity of the cannabis plant was recognised by ancient civilisations and described by emperors in Chinese Pharmacopoeia, documenting more than 2000 years of Chinese agricultural and medicinal plant history (see review by Weston-Green et al., 2021). In the 19th century, the medical use of cannabis dramatically increased in the United States and Europe, but it started to decline during the 20th century due to variable preparations and observed effects in patients (Pisanti and Bifulco, 2017). Nowadays, the claims of therapeutic uses of cannabis are once again coming to the forefront of medicine, and with advancing technology, such as brain imaging, our understanding of how cannabis works is improving (Montero-Oleas et al., 2020).

Δ^9^-tetrahydrocannabinol is derived from the decarboxylation of tetrahydrocannabinolic acid and is responsible for the euphoric properties associated with cannabis use (Amin and Ali, 2019). Other phytocannabinoids are synthesised in the plants at different proportions depending on the chemovar of the plants, for example, cannabidiol (CBD) and cannabidivarin (CBDV; Turner et al., 2017). CBD is well known and has been widely studied for its potential therapeutic benefits in psychiatric and other disorders (see Crippa et al., 2018 for an overview). For example, Leweke et al. showed improvement from baseline in the Positive and Negative Syndrome Scale (PANSS) and the Brief Psychiatric Rating Scale scores in patients with schizophrenia (drug-free for 3 days) after 14 and 28 days of treatment with CBD (Leweke et al., 2012). Similarly, in a randomised double-blind controlled trial, CBD, as an adjunctive treatment in patients with schizophrenia, resulted in a reduction in the PANSS positive subscale and a rating of “improved” by their clinician on the Clinical Global Impressions Scale, with non-significant improvements in Global Assessment of Functioning scale and the Brief Assessment of Cognition in Schizophrenia score (McGuire et al., 2018).

Less is known about the n-propyl analogue of CBD and CBDV. CBDV has been proposed for the treatment of different neurological diseases and has been previously tested in animal models for epilepsy, Rett syndrome, autism, and Fragile X syndrome (Hill et al., 2013; Premoli et al., 2023; Vigli et al., 2018; Zamberletti et al., 2019b). Its mechanism of action is unknown, but CBDV is reported to act as an antagonist at the G protein-coupled receptor 55 (GPR55) receptor (Turner et al., 2017) and lacks affinity for CB1 and CB2 receptors (Hill et al., 2013). More recently, cannabinoids (including CBDV) were demonstrated to exhibit moderate inhibitory effects on the activities of acetylcholinesterase and butyrylcholinesterase enzymes, which may contribute to their modulatory capabilities on the cholinergic system (Puopolo et al., 2022). Recent studies have also reported the effects of CBDV on cognition and social deficits in animal models. Sub-chronic administration of CBDV in *Mecp2* mutant mice, a model that has translational relevance to Rett syndrome, rescued the recognition memory deficit in novel object recognition (NOR; Zamberletti et al., 2019a) and sociability in the three-chamber social test (Vigli et al., 2018). Furthermore, CBDV (20 mg/kg/day, postnatal day (PND)35–PND56, intraperitoneally (i.p.)) ameliorated social behaviour deficits induced by prenatal valproic acid exposure in rats, a model of translational relevance to autism (Zamberletti et al., 2019b). Early administration of CBDV (20/100 mg/kg/day, PND22–PND57, i.p.) prevented NOR and social interaction (SI) deficits in the *Fmr1* knockout mouse model of Fragile X syndrome (Premoli et al., 2023). It is clear that evidence shows that CBDV has benefits in cognition and social impairments in other rodent models of neuropsychiatric disorders; however, it has not yet been tested in a rodent model relevant to schizophrenia.

The aetiology of schizophrenia is complex. Genome-wide association studies have identified hundreds of risk gene variants (Trubetskoy et al., 2022), which interact with environmental risk factors during neurodevelopment to increase an individual’s risk of developing schizophrenia (Wahbeh and Avramopoulos, 2021). Finding common dysregulated systems in this diverse population may improve the models we use to model schizophrenia. A disruption in the glutamatergic system is thought to be one of the key drivers of schizophrenia pathophysiology (Glausier and Lewis, 2017), most likely in a sub-population of the most severely affected patients (Tamminga and Clementz, 2020). Consistent with the glutamatergic theory, our group and others have validated a robust animal model of cognitive and social behaviour impairment in schizophrenia through sub-chronic administration of the *N*-methyl-d-aspartate receptor antagonist phencyclidine (PCP), which disrupts glutamatergic neurotransmission (see Neill et al., 2010 for a review). Of particular relevance to this study, we have shown robust deficits in NOR, reversal learning (RL), and SI tasks accompanied by neurobiological deficits of relevance to schizophrenia pathophysiology (Cadinu et al., 2018; Neill et al., 2014). Deficits in this preclinical model are attenuated by atypical new-generation antipsychotics such as clozapine but not by typical first-generation antipsychotics such as haloperidol, suggesting this model provides a reliable model for testing the efficacy of novel compounds to treat specific cognitive domains and social dysfunction relevant to schizophrenia (Grayson et al., 2007; Idris et al., 2010; Rajagopal et al., 2014). Indeed, there is evidence that some atypical antipsychotics can alleviate negative and cognitive symptoms in some patients (Meltzer and Gadaleta, 2021). Here, we use the sub-chronic phencyclidine (scPCP) model to evaluate the response of animals treated with CBDV in two domains of cognition disrupted in schizophrenia patients (recognition memory and problem solving/reasoning), that is, the NOR paradigm and operant RL test. We then investigated the ability of CBDV to attenuate the SI deficits in this model, an aspect of negative symptoms of schizophrenia.

## Methods

### Animals

A total of 170 experimental and 60 conspecific female Lister Hooded rats (Charles River, UK), weighing 202–285 g on arrival, were used. Rats were housed in groups of five, under standard housing conditions, in individually ventilated cages with two levels (GR1800 Double-Decker Cage, Techniplast, UK) maintained at a constant temperature (21°C ± 2°C) and humidity (45% ± 10%) under a standard 12-hour light: dark cycle (lights on 7:00 a.m.); all testing was carried out in the light phase. For the NOR (*N* = 60) and SI (*N* = 60 test rats and *N* = 60 conspecifics) studies, food and water were available ad libitum. For the RL studies (*N* = 50), water was available ad libitum, but food was restricted to approximately 10 g per rat per day to maintain their body weight at 95% of free-feeding weight. Female rats were used as they have consistently demonstrated reliable performance in our laboratory in a variety of cognitive tests at all stages of the oestrus cycle (McLean et al., 2009; Sutcliffe et al., 2007) and robust deficits following our scPCP dosing (Cadinu et al., 2018; Neill et al., 2010, 2014). In our experience, using males does not result in the same robust phenotype, possibly due to the longer half-life observed in female rats, which is attributed directly to their decreased ability to metabolise the drug (Shelnutt et al., 1999; for further discussion of the use of female rats, see Neill et al., 2016). All experiments were carried out in accordance with the Animals Scientific Procedures Act 1986 and were approved by the University of Manchester AWERB (Animal Welfare & Ethical Review Board).

### Drugs

**Figure 1.**
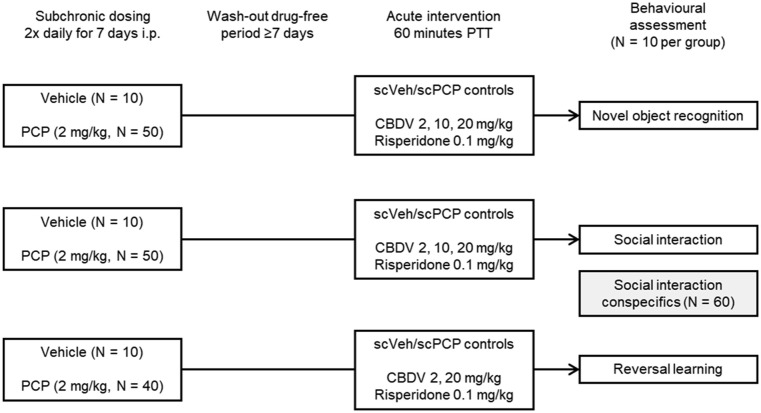
Study design and treatment groups.

Rats were randomly assigned to be pre-treated with either 2.0 mg/kg PCP (Sigma, UK) dissolved in 0.9% saline or vehicle (0.9% saline). PCP and vehicle rats were injected i.p. twice a day, with a mean difference in time between the first and second dose of 6 hours on each day, for 7 days as per our standard dosing regimen (see Cadinu et al., 2018; Neill et al., 2010, 2014; Figure 1). In the NOR and SI studies, rats were tested following a 7-day drug-free period. The RL study tested rats after 28 days due to the extra training required. Despite different washout periods, the rats should be comparable as PCP has a short half-life (around 3.4 hours) and behavioural and relevant pathological changes are detected after different washout durations (Landreth et al., 2020; McKibben et al., 2010; Redrobe et al., 2012; Shelnutt et al., 1999).

Plant-derived highly purified (⩾96.5%) CBDV (GW Research Ltd., part of Jazz Pharmaceuticals) was dissolved in 2:1:17 (ethanol: Cremophor EL: saline 0.9%) and administered via the i.p. route in a volume of 5 ml/kg. CBDV (2, 10, and 20 mg/kg) or vehicle (ethanol: Cremophor EL: saline 0.9%) was administered 60 minutes prior to testing (Figure 1). The initial dose range and pretreatment time of CBDV for NOR studies were chosen from previous studies in other laboratories (Almeida et al., 2013; Deiana et al., 2015; Malone et al., 2009). The narrower dose range of CBDV used in the RL task was chosen based on efficacy in the previous NOR and SI tests. A low, non-D2 receptor blocking dose of the atypical antipsychotic risperidone (0.1 mg/kg; Sigma) was used as a positive control and was dissolved in a minimum volume of acetic acid, made up to volume with 0.9% saline and pH adjusted to 6 with 0.1 M NaOH. Risperidone was administered via the i.p. route in a volume of 1 ml/kg 60 minutes prior to testing. The dose and pretreatment time of risperidone were based on previously generated data (Neill et al., 2016). In all studies, scPCP-treated rats were assigned acute drug administration using a Latin Square design.

## Behavioural tests

### NOR paradigm

The NOR paradigm was performed as previously described in detail (Neill et al., 2016). Briefly, rats were habituated to the empty test chamber for 30 minutes the day before testing. The apparatus consists of an open box made of Plexiglas^®^ (52 cm L; 52 cm W; 31 cm H). The walls of the box were black, and the white floor was divided into nine identical squares (17.3 cm × 17.3 cm). On the test day, scPCP-treated rats received CBDV, risperidone or vehicle as described above. The task consisted of two, 3-minute trials separated by a 1-minute inter-trial interval (ITI). Numerous studies have demonstrated that scPCP-treated rats show pronounced impairments in short-term recognition memory when tested with a 1-minute delay (Grayson et al., 2014; Hjorth et al., 2020; Neill et al., 2016). This likely reflects the limited capacity of the perirhinal cortex (PRC), which processes object recognition but rapidly loses this information over time (Antunes and Biala, 2012). Supporting this, neuroimaging studies in our scPCP model have shown reductions in PRC volume in scPCP-treated rats, which correlated with poor NOR performance (Doostdar et al., 2019). In the acquisition trial, animals were exposed to two identical objects. During the ITI, rats were removed from the box and were individually placed into a small holding box for 1 minute. In the retention trial, a novel object and a new (to avoid olfactory trails), now visually familiar object, were placed in the box with the rat. The NOR box was cleaned with 70% ethanol between acquisition and retention to remove any olfactory traces. Object exploration was defined as the rats sniffing, licking or touching the objects with forepaws whilst sniffing but not by leaning against, turning around, standing or sitting on the objects (Grayson et al., 2007). Behaviour was video recorded (NITEHAWK 8 channel digital video recorder) for subsequent analysis by an experimenter blinded to treatment who had not performed the experiment. Object exploration times in each trial were scored manually. Line crossings were measured as an indicator of locomotor activity by counting the total number of sectors in the box (i.e. lines) crossed during the acquisition and retention trials (Neill et al., 2016). Behavioural scoring underwent random quality control checks by a second scorer to maintain consistency and ensure continuity. Animals that did not explore any of the four objects across acquisition or retention for more than 1 second were excluded from the final analysis.

### RL paradigm

The RL paradigm was performed as described previously (Neill et al., 2016). Briefly, rats were habituated to the RL chambers in pairs (day 1) and then on their own (days 2 and 3). This initial habituation was for 20 minutes each day. Rats were trained to respond for a food reward (45 mg Noyes pellets, PJ Noyes Company Inc.; Sandown Chemical Ltd., Kingston upon Thames, UK) on a fixed ratio 1 schedule of reinforcement so that pressing either of the two active levers delivered a food pellet. Once rats had acquired a lever-pressing response, they were trained to press either the left or right lever (only one was active) for food delivery. Each session (one session per rat per day) lasted 20 minutes, and rats were trained 5 days per week. The active lever varied daily using a pseudorandom Gellerman schedule (Gellermann, 1933). This phase of training lasted approximately 2 weeks. Next, rats were trained to press either the left or right lever for food delivery according to the presence or absence of a visual cue (LED light above the lever). Sessions were terminated after 128 total responses (approximately 30 minutes). Rats were trained once daily, 5 days per week, until they reached ⩾90% correct responding, with each active lever on ⩾3 successive days (generally achieved within 2 weeks). After which rats were trained similarly on the opposite reward contingency (i.e. if the rat had learned to press the lever with a cued light above it for a reward, it now had to learn that the opposite contingency, a lever with no cue light, was rewarded). After completion of RL training, rats were treated sub-chronically either with PCP or vehicle as previously described. RL sessions were discontinued during this treatment period and the subsequent 7-day drug-free period to prevent the development of an association between PCP treatment and the reinforcement contingencies of the RL task and to ensure that PCP-induced deficits were enduring and not related to acute PCP withdrawal. The day before the RL session, rats underwent a full 30-minute training session to ensure stable responding (⩾90% correct responding). In the initial phase of the RL session, the reward contingency was the same as the previous training session, and the test was terminated at 5 minutes or when the animal had earned 20 food pellets. Following the initial phase, where the reward contingency was the same as the previous training session, the rat remained in the test chamber for a 2-minute time-out when the house light was extinguished to cue that the rule was about to change. In the reversal phase, the reward contingency was reversed, and the test was repeated as described above. In general, animals underwent four to six reversal task sessions prior to beginning the drug studies to ensure a stable level of performance, measured by the number of correct responses, in both phases of the task. On the test day, before the initial phase of the RL test, scPCP-treated rats received CBDV, risperidone or vehicle as described above. Rats that failed to press any lever in the initial or reversal phase of the task were excluded.

### SI paradigm

The SI test was performed in the same test chamber used and described for the NOR studies. Test rats were habituated the day before the test to the empty arena. Rats were paired with a weight-matched (within 20 g), untreated, unfamiliar conspecific rat from a different cohort but housed in the same room and conditions. On the test day, scPCP-treated rats received CBDV, risperidone or vehicle as described above. Pairs of rats were simultaneously placed in opposite corners of the test arena, released and behaviour video recorded for a period of 10 minutes. A glass bottle (13 cm H) filled with water was also placed in the centre of the arena. Sniffing of this object was scored as a control behaviour to determine any differences in the interaction of the test animal with an unfamiliar animal as opposed to an unfamiliar object. Recordings were manually scored using behavioural scoring software (Hindsight; Scientific Programming Services, Wokingham, UK) by a trained experimenter blinded to the treatment groups. Recorded behaviours, including investigative social exploration (sniffing any part of the conspecific’s body), object exploration (investigation of the unfamiliar object placed in the centre of the arena) and line crossings (as previously described for NOR), were analysed. Animals that did not explore either the conspecific or the object for more than 1 second were excluded from the final analysis.

### Statistical analysis

All data sets were subjected to D’Agostino and Pearson’s normality test. For the NOR exploration times, data were analysed with a two-way repeated measures ANOVA where the object (left and right or familiar and novel) was the repeated measure and the treatment group was the between-subjects factor. Multiple comparison testing with Sidak’s correction was conducted to compare time spent at familiar and novel objects for each treatment group, where appropriate. For main treatment effects, all groups were compared to scPCP controls using Sidak’s multiple comparison testing. The discrimination index (DI) was calculated by finding the difference between time spent at the novel and familiar object, divided by the total exploration time of the familiar and novel object. For DI, RL, and SI data, normally distributed data were analysed by one-way ANOVA, followed by pairwise comparisons with Dunnett’s correction where appropriate, comparing all groups to scPCP-treated rats. Non-normally distributed data were analysed by Kruskal–Wallis, followed by pairwise comparisons with Dunn’s correction where appropriate, comparing all groups to scPCP-treated rats. Significance was accepted when *p* < 0.050 was achieved. All analyses were performed in GraphPad Prism V10.3.1.

## Results

### The effect of CBDV on cognitive and social deficits in the scPCP model

#### CBDV attenuated recognition memory deficits in NOR

In the acquisition phase, there was no effect of the object (*F*_[1, 50]_ = 1.229; *p* = 0.273) or the object by treatment interaction (*F*_[5, 50]_ = 0.420; *p* = 0.833). The treatment had a significant effect on overall exploration time (*F*_[5, 50]_ = 2.448, *p* = 0.046).

**Figure 2.**
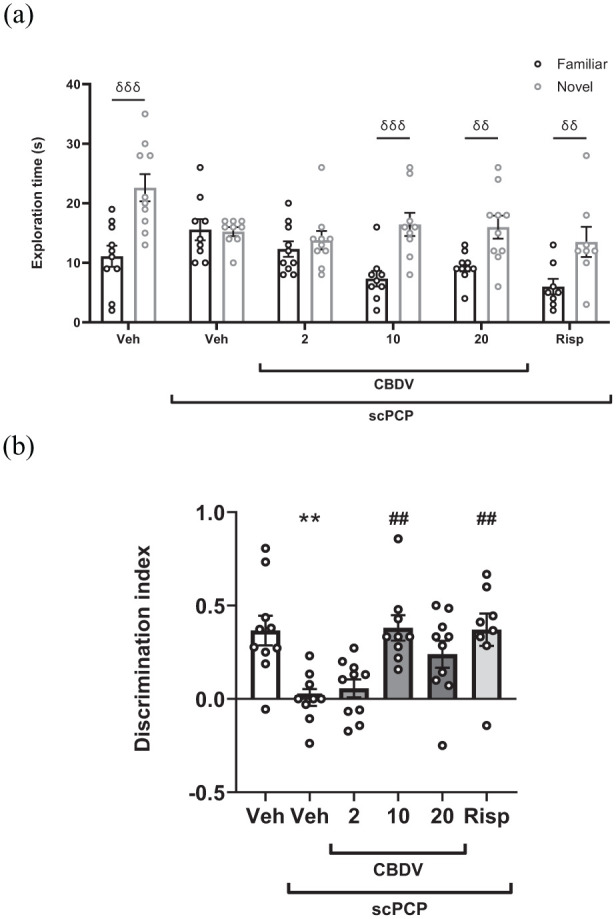
(a) Mean exploration time (s) of familiar and novel objects in the 3 minutes retention trial following acute treatment with CBDV (2, 10, and 20 mg/kg, i.p., PTT 60 minutes) and Risp (0.1 mg/kg, i.p., PTT 60 minutes) in scPCP-treated rats (2 mg/kg, i.p. twice daily for 7 days, followed by a 7-day drug-free period). Vehicle-treated rats received 0.9% saline, i.p., twice daily for 7 days, followed by a 7-day treatment-free period. Data were expressed as mean ± SEM (*N* = 8–10 per group). Data were analysed with a two-way repeated measures ANOVA, followed by pairwise comparisons of familiar versus novel with Sidak’s correction. (b) The effect of acute treatment with CBDV (2, 10, and 20 mg/kg, i.p., PTT 60 minutes) and risperidone (0.1 mg/kg, i.p., PTT 60 minutes) in scPCP-treated rats on DI. Data were expressed as the mean ± SEM (*N* = 8–10 per group) and were analysed by one-way ANOVA followed by Dunnett’s multiple comparison tests comparing all groups to scPCP/Veh. Significant difference between time spent at familiar and novel objects: δδ*p* < 0.010, δδδ*p* < 0.001. Significant difference from Veh group: **p < 0.010. Significant difference from scPCP/Veh: ##*p* < 0.010. SEM: standard error of the mean; CBDV: cannabidivarin; PTT: pretreatment time; scPCP: sub-chronic phencyclidine; Risp: risperidone; i.p.: intraperitoneally; DI: discrimination index.

In the retention trial, there was a significant effect of the object (*F*_[1, 50]_ = 62.890, *p* < 0.001), treatment (*F*_[5, 50]_ = 3.143, *p* = 0.015) and the object by treatment interaction (*F*_[5, 50]_ = 6.138, *p* < 0.001). Multiple comparison testing revealed that there was a significant difference in the time spent exploring the familiar and novel objects in the vehicle-treated animals (*p* < 0.001) but not in the scPCP group (*p* > 0.999). The scPCP-induced deficit in NOR was attenuated following treatment with CBDV at 10 and 20 mg/kg (*p* < 0.001 and *p* = 0.003) and the positive control risperidone (*p* = 0.003) but not CBDV at 2 mg/kg (*p* = 0.955; Figure 2(a)).

Treatment had a significant effect on exploration time in the acquisition and retention trials. In both trials, this was due to reductions in object exploration time in rats treated with risperidone compared to scPCP controls (Acquisition: *p* = 0.045; Retention: *p* = 0.015; Table 1). These effects occurred in the absence of any locomotor effects (Line crossings: (F[5, 50] = 1.839); *p* = 0.122; Table 2).

**Table 1.** Effect of acute treatment of CBDV (2, 10, and 20 mg/kg, i.p., PTT 60 minutes) and risperidone (0.1 mg/kg, i.p., PTT 60 minutes) on average exploration time in Acq. and Ret. trials of the NOR task, shown as mean ± SEM (*N* = 8–10).

NOR Trial	scVeh/Veh	scPCP/Veh	2 mg/kg CBDV	10 mg/kg CBDV	20 mg/kg CBDV	Risp
Acq.	11.4 (0.70)	13.9 (0.78)	14.7 (0.20)	12.6 (1.11)	12.9 (1.30)	8.3 (0.44)*
Ret.	16.9 (5.75)	15.4 (0.17)	13.1 (0.75)	11.9 (4.56)	12.7 (3.35)	9.8 (3.75)*

The main effects of treatment were assessed with a two-way repeated measures ANOVA followed by Sidak’s correction comparing average object exploration to scPCP controls. Significant difference from scPCP/Veh group: *p < 0.050.

NOR: novel object recognition; SEM: standard error of the mean; CBDV: cannabidivarin; PTT: pretreatment time; scPCP: sub-chronic phencyclidine; Acq.: acquisition; Ret.: retention; LX: line crossings; i.p.: intraperitoneally.

**Table 2.** Effect of acute treatment of CBDV (2, 10, and 20 mg/kg, i.p., PTT 60 minutes) and risperidone (0.1 mg/kg, i.p., PTT 60 minutes) on the number of LX in the NOR task, shown as mean ± SEM (*N* = 8–10).

Behaviour	scVeh/Veh	scPCP/Veh	2 mg/kg CBDV	10 mg/kg CBDV	20 mg/kg CBDV	Risp
LX	79.6 (7.63)	79.8 (3.36)	68.6 (4.46)	79.8 (5.21)	66.8 (4.31)	63.9 (6.20)

Data were analysed with a one-way ANOVA.

NOR: novel object recognition; SEM: standard error of the mean; CBDV: cannabidivarin; PTT: pretreatment time; scPCP: sub-chronic phencyclidine; LX: line crossings; i.p.: intraperitoneally.

Treatment had a significant effect on the DI (*F*_[5, 50]_ = 5.963, *p* < 0.001). Multiple comparison tests showed a significant reduction in DI in scPCP-treated rats compared to Veh controls (*p* = 0.002), which 10 mg/kg CBDV (*p* = 0.002) and risperidone (*p* = 0.004) reversed. There was no improvement from scPCP in rats treated with 2 mg/kg (*p* = 0.980) and 20 mg/kg (*p* = 0.074; Figure 2(b)). Four rats were excluded from this study due to failure to explore (1 scPCP, 1 CBDV 10 mg/kg and 2 risperidone).

#### CBDV significantly attenuated the scPCP deficit in RL

**Figure 3.**
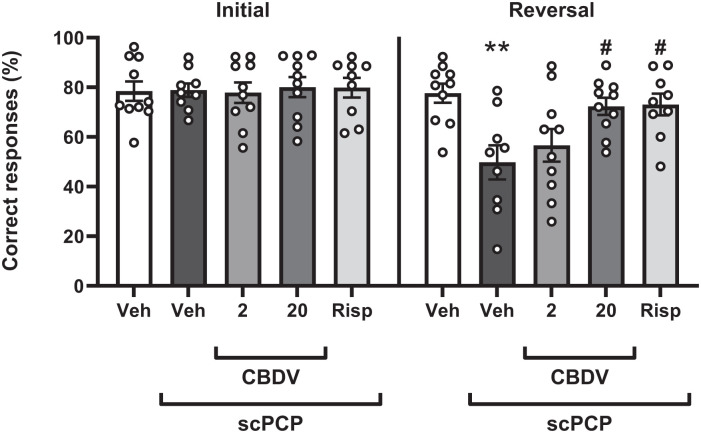
Percentage of correct responding in the initial and reversal phase of the RL test following treatment with vehicle, CBDV (2 and 20 mg/kg, i.p., PTT 60 minutes) or risperidone (0.1 mg/kg, i.p., PTT 60 minutes) in scPCP-treated rats (2 mg/kg, i.p. twice daily for 7 days, followed by a 28-day drug-free period). Vehicle-treated rats received 0.9% saline, i.p., twice daily for 7 days, followed by a 28-day treatment-free period. Data were expressed as mean ± SEM (*N* = 9–10 per group) and were analysed by one-way ANOVA followed by Dunnett’s multiple comparison tests comparing all groups to scPCP/Veh. Significant difference from Veh group: **p < 0.010. Significant difference from scPCP/Veh: #*p* < 0.050. SEM: standard error of the mean; CBDV: cannabidivarin; PTT: pretreatment time; scPCP: sub-chronic phencyclidine.

During the initial phase, the reward contingency is the same as the training session. Here, the percentage of correct responses was unaffected by treatment (*F*_[4, 43]_ = 0.062; *p* = 0.993; Figure 3). After the rule switched to the opposite contingency in the reversal phase, there was a significant effect of treatment (*F*_[4, 43]_ = 5.287; *p* = 0.002; Figure 3). Posthoc analysis showed a significant reduction in percentage of correct responding in the scPCP group (*p* = 0.002) compared to vehicle. This was significantly attenuated by CBDV at 20 mg/kg (*p* = 0.013) and the positive control, risperidone (*p* = 0.013), but not by CBDV at 2 mg/kg (*p* = 0.752; Figure 3). To confirm that the drug did not affect the rats’ ability to complete the task, the total number of lever presses in each phase was compared. Here, there was no treatment effect on lever presses in the initial (*H*_[4]_ = 1.119, *p* = 0.891; Table 3) or reversal phase (*F*_[4, 43]_ = 0.507, *p* = 0.840; Table 3). In this study, two animals (one scPCP and one risperidone) were excluded due to failure to pass training thresholds after scPCP dosing.

**Table 3.** Effect of acute treatment of CBDV (2 and 20 mg/kg, i.p., PTT 60 minutes) and risperidone (0.1 mg/kg, i.p., PTT 60 minutes) on the total number of lever presses in the initial and the reversal phases of the RL task, shown as mean ± SEM (*N* = 9–10).

RL phase	scVeh/Veh	scPCP/Veh	2 mg/kg CBDV	20 mg/kg CBDV	Risp
Initial	26.2 (0.53)	26.2 (0.36)	26.1 (0.23)	26.2 (0.39)	26.3 (0.17)
Reversal	26.4 (0.16)	26.7 (0.24)	26.4 (0.22)	26.1 (0.23)	26.4 (0.24)

Data were analysed with a one-way ANOVA.

SEM: standard error of the mean; CBDV: cannabidivarin; PTT: pretreatment time; scPCP: sub-chronc phencyclidine; RL: reversal learning; i.p.: intraperitoneally.

#### CBDV attenuated behavioural deficits in SI

**Figure 4.**
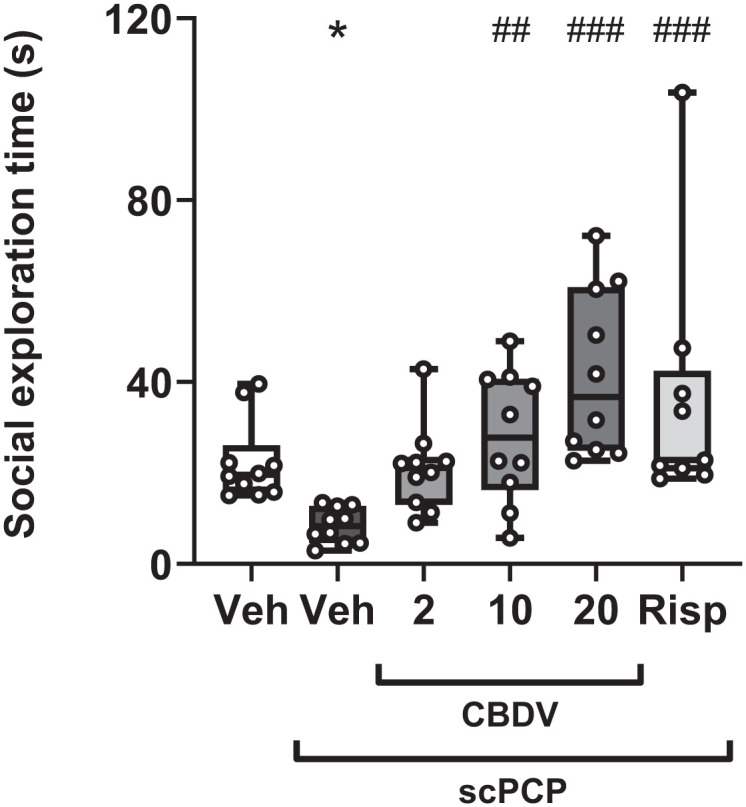
Effect of acute treatment of CBDV (2, 10, and 20 mg/kg, i.p., PTT 60 minutes) and risperidone (0.1 mg/kg, i.p., PTT 60 minutes) on social exploration in scPCP-treated rats (2 mg/kg, i.p. twice daily for 7 days, followed by a 7-day drug-free period). Vehicle-treated rats received 0.9% saline, i.p., twice daily for 7 days followed by a 7-day treatment-free period. Data were shown as median with interquartile ranges; whiskers denote the minimum and maximum points (N = 9–10 per group). Data were analysed by Kruskal–Wallis, followed by pairwise comparisons with Dunn’s correction. Significant difference from Veh group: *p < 0.050. Significant difference from scPCP/Veh: ##*p* < 0.010 and ###*p* < 0.001. CBDV: cannabidivarin; PTT: pretreatment time; scPCP: sub-chronic phencyclidine.

The social exploration time was significantly altered by treatment groups (*H*_[5]_ = 26.300; *p* < 0.001; Figure 4). Posthoc analysis indicated that social exploration was reduced in the scPCP group compared to vehicle controls (*p* = 0.048) and that this was attenuated by CBDV at 10 and 20 mg/kg (*p* = 0.002 and *p* < 0.001) and by the positive control, risperidone (*p* < 0.001). CBDV at 2 mg/kg trended towards a recovery of social exploration (*p* = 0.052; Figure 4). Whilst treatment did not affect object exploration (*H*_[5]_ = 5.798; *p* = 0.326; Table 4), it significantly altered the number of line crossings (*F*_[5, 53]_ = 2.851; *p* = 0.024), which may be due to risperidone significantly reducing the number of line crossings compared to scPCP (*p* = 0.033; Table 5). In this study, one risperidone rat was culled due to a health issue unrelated to the experimental procedure.

**Table 4.** Effect of acute treatment of CBDV (2, 10, and 20 mg/kg, i.p., PTT 60 minutes) and risperidone (0.1 mg/kg, i.p., PTT 60 minutes) on object exploration in the SI task.

Behaviour	scVeh/Veh	scPCP/Veh	2 mg/kg CBDV	10 mg/kg CBDV	20 mg/kg CBDV	Risp
Obj	43.6 (2.67)	42.9 (4.64)	49.3 (3.51)	56.7 (7.36)	54.0 (4.70)	42.4 (4.12)

Shown as mean ± SEM (*N* = 9–10). Data were analysed with a Kruskal–Wallis test.

SEM: standard error of the mean; CBDV: cannabidivarin; PTT: pretreatment time; scPCP: sub-chronic phencyclidine; i.p.: intraperitoneally; SI: social interaction.

**Table 5.** Effect of acute treatment of CBDV (2, 10, and 20 mg/kg, i.p., PTT 60 minutes) and risperidone (0.1 mg/kg, i.p., PTT 60 minutes) on the number of LX in the SI task, shown as mean ± SEM (*N* = 9–10).

Behaviour	scVeh/Veh	scPCP/Veh	2 mg/kg CBDV	10 mg/kg CBDV	20 mg/kg CBDV	Risp
LX	114.8 (3.24)	126.3 (6.28)	130.1 (6.35)	122.3 (7.54)	127.6 (6.28)	102.2 (5.61)*

Data were analysed with a one-way ANOVA followed by Dunnett’s multiple comparison test comparing all groups to scPCP controls.

Significant difference from the scPCP/Veh group: *p < 0.050.

SEM: standard error of the mean; CBDV: cannabidivarin; PTT: pretreatment time; scPCP: sub-chronic phencyclidine; i.p.: intraperitoneally; LX: line crossings; SI: social interaction.

## Discussion

In this study, we investigated the ability of CBDV to attenuate scPCP-induced cognitive and social deficits in female Lister Hooded rats. Recently, CBDV has been proposed as a candidate for the treatment of psychiatric disorders, including schizophrenia, but its efficacy is still unknown. Despite this, evidence from phase 2 clinical trials suggests that CBDV is safe as an add-on therapy for epilepsy (Brodie et al., 2021). The NOR and RL tasks assessed recognition memory and cognitive flexibility, two cognitive domains affected by schizophrenia (Young et al., 2009). Here, we confirm the robust scPCP-induced deficit in NOR and RL, as we have reported previously (Hjorth et al., 2020; Idris et al., 2009; Landreth et al., 2020; McLean et al., 2017). Treatment with CBDV (10 and 20 mg/kg) attenuated the scPCP deficit in the NOR task. In the RL paradigm, CBDV at the highest dose tested (20 mg/kg) improved the cognitive deficit in RL.

Negative symptoms remain one of the unmet clinical needs for schizophrenia patients, and we and others have consistently demonstrated scPCP-induced social behaviour deficits (Gururajan et al., 2010; Neill et al., 2014). In the SI test, scPCP produced a reduction in social exploration, which was restored by CBDV at 10 and 20 mg/kg, suggesting a specific effect to ameliorate scPCP-induced social behaviour deficits. We did not observe any effect of CBDV on the total number of line crossings or object exploration.

Unusually, risperidone decreased exploration times in the NOR task and line crossings in the SI task. We have not seen altered locomotor activity with the same dose of risperidone previously (Hjorth et al., 2020; Neill et al., 2016). However, this does not seem to effect the performance of the animals in these tasks, that is, a distinction between novel and familiar objects is still seen in the NOR task and exploration of a conspecific is increased following risperidone administration in the SI task, thereby confirming the efficacy of risperidone in our model as we have seen previously (Neill et al., 2016). Although controversial, antipsychotics like risperidone are frequently employed as “positive controls” in preclinical studies, despite their limited efficacy in addressing cognitive and negative symptoms in patients (Marder and Umbricht, 2023; McCutcheon et al., 2023). We have used this dose of risperidone (0.1 mg/kg) in our previous preclinical studies (Neill et al., 2016). In our experience, this dose produces minimal sedation and fails to counteract amphetamine-induced hyperlocomotion, effects typically observed in drugs with antipsychotic effects (unpublished data). Additionally, this dose of risperidone is insufficient to occupy dopamine D2 receptors at a clinical meaniningful level in animals (Kapur et al., 2003). It is believed that its observed efficacy in these models is more likely attributable to its effects on serotonergic pathways (Neill et al., 2010).

Interpreting the clinical translatability of these preclinical findings remains a major challenge, particularly since most clinical trials evaluating antipsychotics for cognitive and negative symptoms use doses that strongly block D2 receptors. It has long been suggested that the high levels of D2 antagonism, required for antipsychotic efficacy, may obscure any beneficial effects on cognitive and negative symptom domains (Miyamoto et al., 2005). Therefore, the term “positive control” in this context specifically refers to its role within our preclinical framework.

The scPCP regimen has been validated in several laboratories as a reliable model to mimic cognitive and negative symptoms of schizophrenia (Grayson et al., 2007; Gururajan et al., 2010; Neill et al., 2010; Rajagopal et al., 2014; Shahid et al., 2020). The advantage of this model compared to acute PCP administration or other chronic PCP dosing regimens is that testing is conducted in the drug-free state to avoid any direct effect of PCP on behaviour or any direct interaction between PCP and the drug being studied.

CBDV showed efficacy on cognitive and social deficits, demonstrating a dose-dependent effect on novel object exploration in the retention phase of the NOR test and on social exploration behaviour in the SI task. These findings were in female Lister Hooded rats, and the efficacy in males warrants further investigation. The RL experiment used two doses (2 and 20 mg/kg) of CBDV, carefully selected from the NOR and SI efficacy studies. The reduced number of doses used in this study was also due to limited animal availability. We acknowledge that this narrow dose range is a limitation which further studies could address.

The mechanism of action for CBDV is poorly understood, but as a propyl analogue of CBD, it may have similar molecular targets. For example, neurotransmitter receptors (adenosine, glycine, opioids, 5-HT1A), GPR55, enzymes (FAAH), ion channels (TRPV1), and transporters (ENT1) have all been implicated in the therapeutic effects of CBD (Gray and Whalley, 2020; Ibeas Bih et al., 2015; Turner et al., 2017). Indeed, a recent review highlights that CBD and CBDV are agonists at TRPA1, TRPV1, and TRPV2, an inverse agonist at GPR6 and an antagonist at GPR55 (Alves et al., 2020). Certainly, there is evidence indicating adenosine dysfunction (Gomes et al., 2011) and the involvement of TRPV1 receptors (Chahl, 2007) in schizophrenia, with preclinical studies suggesting adenosine, TRPV1 receptors and GPR55 receptors play a role in dopamine and/or glutamate release (Almeida et al., 2014; Carrier et al., 2006; Fredholm et al., 2005; Kramar et al., 2017; Ligresti et al., 2016; Petrovszki et al., 2014; Popoli et al., 1994; Sills et al., 1999; Solinas et al., 2002; Thompson et al., 1993; Wu and Saggau, 1997).

Research utilising animal models has undoubtedly played a crucial role in advancing our understanding of complex disorders like schizophrenia (Uliana et al., 2023). These models have been continuously refined to assess relevant symptom domains and further aid our understanding of the underlying molecular mechanisms (Heinssen et al., 2024). However, we have yet to see the benefits for patients, in relation to new treatments for the unmet clinical needs (i.e. cognitive and negative symptoms). A number of recent failures in phase 3 clinical trials despite promising preclinical (and often very promising phase 2) data have inevitably led to further scrutiny of animal models and questions about their translation (Nutt, 2025).

It is widely recognised that schizophrenia is inherently a human disorder and, as such, there will be an unavoidable limit to predicting clinical outcomes. However, efforts to utilise models with improved neurobiological validity alongside preclinical tests of efficacy that map onto those used clinically will be important in developing novel treatments with potential for efficacy in humans (see Spark et al., 2022; Young, 2023; Young and Byrnes, 2022, for detailed reviews). Although our findings support some therapeutic potential of CBDV in ameliorating cognitive impairments associated with schizophrenia, the underlying mechanisms remain to be fully elucidated. Future studies focusing on long-term intervention with CBDV, combining translationally relevant behavioural and molecular readouts, can bring greater insight into drug action.

In summary, these promising, albeit preliminary, results support further investigations of CBDV in the treatment of cognitive and social behaviour deficits in schizophrenia. Further mechanistic studies are required to understand how these phytocannabinoid-mediated effects are produced.
